# Melanoma antigen-specific effector T cell cytokine secretion patterns in patients treated with ipilimumab

**DOI:** 10.1186/s12967-017-1140-9

**Published:** 2017-02-21

**Authors:** Yana G. Najjar, Fei Ding, Yan Lin, Robert VanderWeele, Lisa H. Butterfield, Ahmad A. Tarhini

**Affiliations:** 1 University of Pittsburgh Medical Center, Pittsburgh, PA USA; 2 Biostatistics Facility, University of Pittsburgh Cancer Institute, Pittsburgh, PA USA; 3 University of Pittsburgh Medical Center, Pittsburgh, PA USA; 4 University of Pittsburgh, University of Pittsburgh Cancer Institute, Pittsburgh, PA USA; 5 Division of Hematology-Oncology, Department of Medicine, University of Pittsburgh, University of Pittsburgh Cancer Institute, 5150 Centre Avenue (555), Pittsburgh, PA 15232 USA

**Keywords:** Neoadjuvant, Melanoma, Ipilimumab, Cytokines

## Abstract

**Background:**

In a previously reported study, patients with regionally advanced melanoma were treated with neoadjuvant ipilimumab (ipi) (Tarhini in PLoS ONE 9(2): e87705, 3). Significant changes in circulating myeloid derived suppressor cells (MDSC), regulatory T cells (Treg) and peptide specific type I CD4^+^ and CD8^+^ T cells were noted at week 6 that correlated with clinical outcome. Characterization of antigen-specific effector T cell secreted cytokines may shed insights into ipi associated T cell activation and function.

**Methods:**

Patients were treated with neoadjuvant ipi (10 mg/kg every 3 weeks ×2) administered intravenously before and after surgery. Peripheral blood mononuclear cells (PBMC) that were collected at baseline and week 6 (after ipi) were tested here. Each sample was divided into 5 groups and stimulated with controls or shared melanoma antigen overlapping peptide pools (NY-ESO 1, gp-100, MART-1). Secreted cytokines, chemokines and growth factors were assessed using Luminex. Cytokine expression levels between the 3 antigen groups were compared using the Wilcox rank-sum test.

**Results:**

Seventeen cytokines were differentially expressed with stimulation by each antigen at baseline (p value <0.05): IL1β, MIP1β, IL1RA, VEGF, IL13, IL17, MIP1α, GM-CSF, MCP1, IL5, IL2R, IL4, IL10, IFNγ, TNFα, IL8 and IL2. At week 6, 15 cytokines were differentially expressed (p < 0.05): IL1β, VEGF, G-CSF, HGF, IL13, IL17, GM-CSF, MCP1, IL5, IL7, IL4, IL10, IFNγ, IL8 and IL2. Patients were later clustered based on cytokine expression levels at baseline and at week 6, and recurrence free survival (RFS) was compared. Clear differences in RFS were noted based on cytokine level clustering both at baseline and at week 6: Patients whose PBMCs secreted more cytokines in response to NY-ESO-1 showed a trend towards better RFS.

**Conclusions:**

PBMCs of patients treated with ipi secreted significantly more cytokines, chemokines and growth factors in response to NY-ESO-1 than to gp-100 or MART-1. These cytokines belonged to different functional groups, including inflammatory, type 1, type 2 and regulatory, that warrant further study. Patients whose PBMCs secreted more cytokines (particularly in response to NY-ESO-1) tended to have better RFS, supporting further exploration in terms of therapeutic predictive value.

**Electronic supplementary material:**

The online version of this article (doi:10.1186/s12967-017-1140-9) contains supplementary material, which is available to authorized users.

## Background

Melanoma continues to be a major public health problem in the United States (U.S.), with an estimated 76,380 new cases diagnosed in 2016 and 10,130 deaths [1]. The treatment paradigm for patients with unresectable or metastatic disease has been revolutionized by the approval of novel targeted and immunotherapeutic agents in recent years, but patients with resectable loco-regionally advanced melanoma continue to have poor outcomes. Indeed, the majority of patients with locoregional disease, including lymph node metastases, in transit metastases or satellite lesions will have recurrence after initial resection and develop metastatic disease [2, 3]. The prognosis for patients with stage I disease is excellent, with 5 year survival rates of 97% [4], though with increasing thickness, 5 year survival rates decrease. For example, patients with stage IIC disease have a 5 year survival rate of 53% [4]. Outcomes for patients with stage III melanoma are overall worse, with 5 year survival of 78, 59, and 40% for stage IIIA, IIIB, and IIIC, respectively [4]. Recurrence risk for stage III melanoma also varies: 5-year overall recurrence free survival (RFS) for patients with stage IIIA, IIIB, and IIIC melanoma is 63, 32, and 11%, respectively [5], and the key prognostic factors are nodal involvement and primary features of the tumor [4]. The current standard of care for patients with locoregionally advanced disease is surgery followed by adjuvant high-dose interferon, pegylated interferon or ipilimumab [4, 6]. Because over half of patients with treated clinically detectable locoregional disease eventually develop metastases and die of their disease, there is an urgent need to develop novel approaches to the treatment of stage III melanoma, including neoadjuvant therapy.

In patients with locally advanced disease, neoadjuvant therapy has several potential advantages over adjuvant [3], and has been shown to improve survival outcomes in several malignancies, including esophageal, breast and bladder cancer [7–9]. Moreover, neoadjuvant treatment may facilitate surgery by shrinking tumors prior to surgery, and may decrease the risk of metastasis by eradicating micrometastatic disease sooner rather than later [3].

We have previously reported a single-arm neoadjuvant phase II study in patients with stage IIIB-C melanoma. Patients received high dose ipilimumab (10 mg/kg IV q3 weeks ×4 doses total) bracketing definitive surgery [9]. Ipilimumab is a monoclonal antibody that binds to CTLA-4, which is up-regulated on T cells after activation and functions as a negative regulator of T cell activation. Median PFS was 15.5 months among 29 evaluable patients. In our previous analysis significant changes in circulating regulatory T cells (Treg), myeloid derived suppressor cells (MDSC) and peptide specific type I CD4^+^ and CD8^+^ T cells were observed at 6 weeks that correlated with clinical outcome [9]. A significant increase in circulating Treg was noted at week 6 compared to baseline (p = 0.02), and increased Treg were associated with improved PFS (p = 0.034). A significant decrease in peripheral MDSC was also noted, especially in the monocytic gate (HLA-DR^+^/low, CD14^+^; p < 0.0001), a key MDSC subset, and this was associated with improved PFS (p = 0.03) [9]. Interestingly, and notably in the absence of any antigen specific stimulation, circulating CD4^+^ and CD8^+^ T cells specific for commonly expressed shared melanoma tumor antigens (gp-100, MART-1, NY-ESO-1) were increased in frequency after treatment. Taken together, these data suggest that ipilimumab favorably alters and positively modulates the circulating immune environment, and increases melanoma antigen-specific T cell responses. The characterization of T cell secreted cytokines may shed insights into ipilimumab associated T cell activation and function. Here, we report on differential T cell cytokine secretion at baseline and at week 6 (following systemic therapy with ipilimumab) in response to melanoma lineage (gp-100, MART-1) and cancer testis (NY-ESO 1) antigen stimulation in 13 patients treated with ipilimumab in the neoadjuvant setting.

## Methods

### Patients and ethics

The characteristics of the total patient population were reported in the previously published study [15]. Characteristics of the 13 tested here are shown in Table 1. The study was approved by the University of Pittsburgh Institutional Review Board (IRB; IRB# PRO09010033). All patients had a University of Pittsburgh IRB approved written informed consent obtained. The study was conducted in accordance with the principles expressed in the Declaration of Helsinki. Eligible patients were 18 years or older and had clinically detectable locally and/or regionally advanced melanoma (cutaneous, mucosal or unknown primary).

**Table 1:** Patient demographics and baseline disease characteristics (N = 13 patients)

Variable	No. of patients (%)
Age, years; median	50 (range 40–81)
Cutaneous primary	11 (84)
Mucosal primary	1 (8)
Unknown primary	1 (8)
Gender	
Female	3 (23)
Male	10 (77)
Performance status (ECOG)	
0	10 (77)
1	3 (23)
Stage	
IIIB	1 (8)
IIIC	12 (92)
Tumor mutational status	
BRAF^V600^	6 (46)
NRAS^Q61^	2 (15)
NRAS^R73^	1 (8)
BRAF/NRAS WT	3 (23)
Unknown	1 (8)

*ECOG* Eastern Cooperative Oncology Group

### Study design

Thirty patients were treated with neoadjuvant ipilimumab administered at 10 mg/kg intravenously (I.V.) on day 0 and day 21 followed by surgery at least 6 weeks later. Additional maintenance ipilimumab was offered following surgery, as we have previously reported [10]. Peripheral blood mononuclear cells (PBMC) samples collected and cryopreserved at baseline and 6 weeks were used for this analysis from 13 patients who had either relapsed [3] or not [6] within the first year. PBMCs from each time point were thawed, aliquoted to a concentration of 1 × 10^6^ cells/ml in complete media, and each sample was then separated into 5 samples.

Libraries of 15-mer peptides overlapping by 4 amino acids were constructed (Mimetopes, Minneapolis, MN) to measure CD4^+^ and CD8^+^ T cells specific to tumor antigens (Gp-100, MART-1, NY-ESO-1) in an HLA-unrestricted fashion. PBMC were either [1] unstimulated (negative control), or stimulated with either [2] PMA/inomycin (positive control), [3] a peptide pool consisting of 27 peptides from MART-1, [4] a peptide pool consisting of 163 peptides from gp100, [5] a peptide pool consisting of 43 peptides from NY-ESO-1. Samples were then incubated at 37 °C for 24 h. Samples were centrifuged and the supernatants saved in aliquots at −80 °C in a temperature controlled and monitored freezer. Samples did not thaw before testing. Supernatants were analyzed using the Multiplex Bead-based Luminex human 30-plex Assay cytokine plate, as per standard protocol, and run on a BioPlex system (BioRad). The Cytokine Human 30-Plex Panel for the Luminex platform quantifies GM-CSF, IL-1β, IL-5, IL-4, IL-2, TNF-α, IL-6, RANTES, MIG, VEGF, HGF, EGF, IL-8, IL-17, MIP-1α, IL-10, G-CSF, MCP-1, IL-7, IL-15, IFN-α, IL-2R, IP-10, MIP-1β, Eotaxin, IL-1RA, IL-12 (p40⁄p70) IL-13, FGF-Basic and IFN-γ (Additional file 1: Table S1). Assay controls included the kit standards for standard curves and Multiplex controls (R&D Systems).

## Statistical methods

Expression level of cytokines in response to antigen stimulations were obtained by subtracting the corresponding negative control value from measured values. If the measured value was under the detection limit or lower than the negative control value, it was assigned zero.

At baseline and week 6, Wilcox rank-sum test was used to identify cytokines that had different expression levels (unadjusted p value <0.05) when PBMCs were stimulated with NY-ESO-1 compared to the other two antigens. Expression levels of these cytokines under antigen stimulation at each time point were plotted in a heatmap. For better visualization, non-zero values were truncated and logged.

Hierarchical clustering of patients was then performed using these differentially expressed cytokines as features at each time point. Due to sparsity of data, expression level of cytokines was dichotomized. Both Jaccard distance and 1-Phi coefficient were used as dissimilarity measure with eight different types of linkage to perform the clustering, and the patterns that consistently emerging were presented here.

Based on clustering results, patients were grouped into two clusters. Fisher’s exact test was applied to examine the association between cluster assignment and dichotomized RFS at 1 year. RFS of the two clusters were also compared using Kaplan–Meier plot and log-rank test. Because this is an exploratory study, p values were not adjusted for multiple testing.

## Results

### Patient information

Thirteen patients with available biospecimens were included in this analysis (Table 1). The median age was 50 (range 40–81), with 10 males (77%) and 3 females (23%). The majority of patients (11; 84%) had a cutaneous primary, and 1 (8%) each had mucosal melanoma or melanoma of unknown primary. 10 patients (77%) were entirely asymptomatic, with an ECOG performance status of 0, and 3 patients (23%) had an ECOG of 1. These patients were at high risk of recurrence, with 12 patients (92%) diagnosed as stage IIIC, and 1 patient (8%) had stage IIIB disease. 6 patients (46%) had a BRAF^V600^ mutation. 2 patients (15%) were found to have NRAS^Q61^ mutation, and one had an NRAS^R73^ mutation. 3 patients (23%) were BRAF/NRAS wild type, and one patient’s mutational status was unknown. All patients included in this analysis were treated with neoadjuvant ipilimumab bracketing definitive surgery, as previously reported.

### Cytokine profiles are differentially expressed at baseline in response to antigen stimulation

**Fig. 1:**
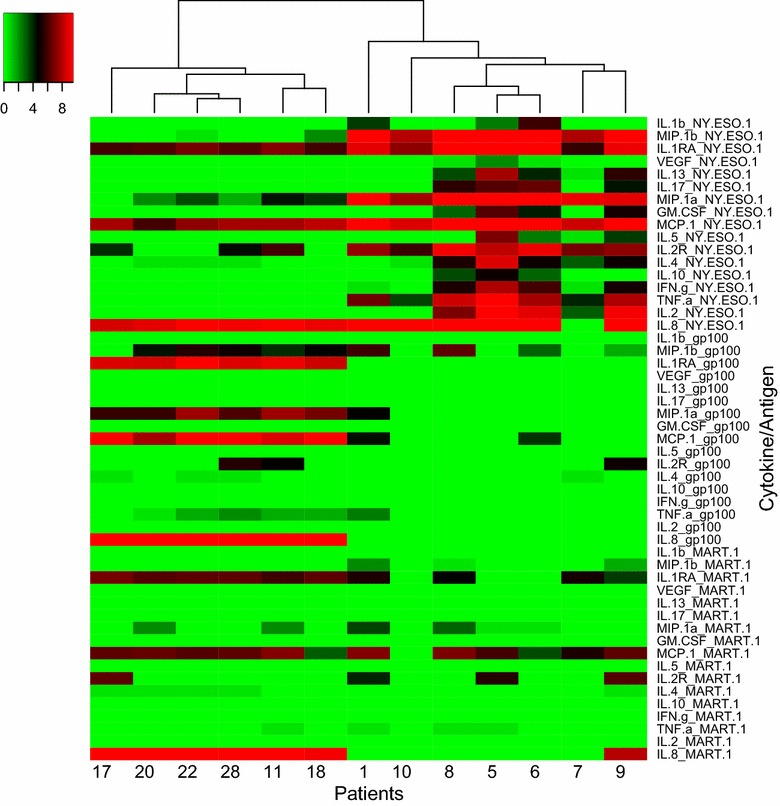
Heatmap of 17 analytes selected at Baseline: Expression pattern of 17 cytokines that respond differently to stimulation with NY-ESO-1 vs. gp-100 and MART-1 at baseline (p value <0.05): IL1β, MIP1β, IL1RA, VEGF, IL13, IL17, MIP1α, GM-CSF, MCP1, IL5, IL2R, IL4, IL10, IFN-γ, TNFα, IL8 and IL2. Patients were clustered into 2 groups: seven patients in cluster 1: 1, 5, 6, 7, 8, 9, and 10 tend to have better RFS and they also have higher cytokine expression when stimulated with NY-ESO-1

To determine the broad secreted protein expression pattern of the shared melanoma antigen specific T cells in the circulation of these patients, and how they might be impacted by ipi, 30 cytokines, chemokines and growth factors in the supernatant of stimulated T cells were tested at baseline and at week 6 using the Human 30-Plex panel for Luminex. At baseline, prior to any treatment with ipilimumab, 17 cytokines were found to have higher expression levels when stimulated with NY-ESO-1 versus gp-100 or MART-1. On stimulation with NY-ESO1, expression of IL-1β, MIP1-β, IL1-RA, VEGF, IL-13, IL-17, MIP1-α, GM-CSF, MCP-1, IL-5, IL2-R, IL-4, IL-10, IFN-γ, TNF-α, IL-8 and IL-2 was significantly higher (p value <0.05 for each) (Fig. 1).

### Cytokine profiles are differentially expressed at week 6 in response to antigen stimulation

**Fig. 2:**
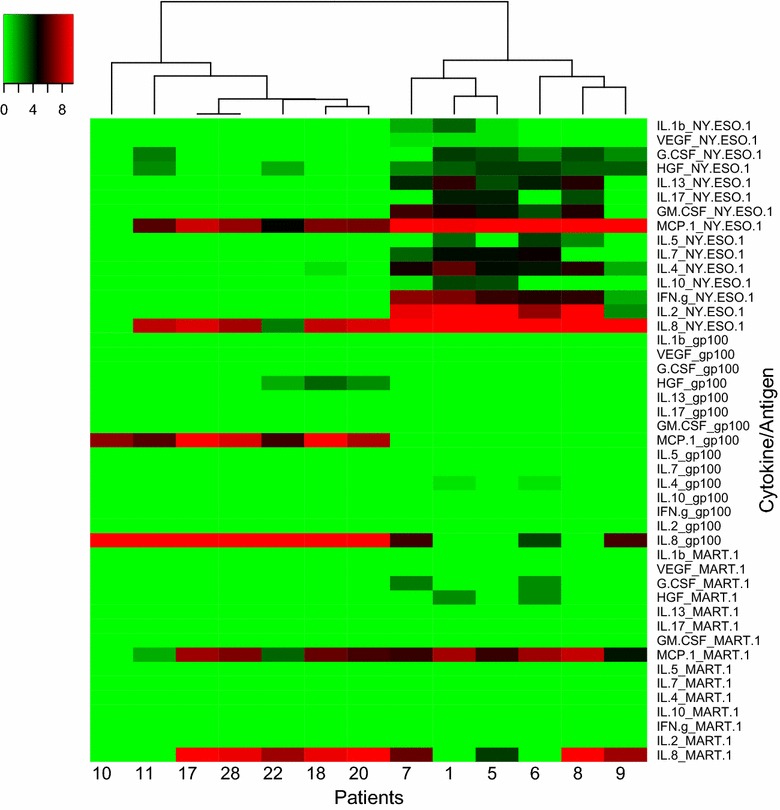
Heatmap of 15 analytes selected Week 6: Expression pattern of 15 cytokines that respond differently to stimulation with NY-ESO-1 versus gp-100 and MART-1 at week 6 (p value <0.05): IL-1β, VEGF, G-CSF, HGF, IL-13, IL-17, GM-CSF, MCP-1, IL-5, IL-7, IL-4, IL-10, IFN-γ, IL-8 and IL-2 Patients were clustered into 2 groups: six patients in cluster 1: 1, 5, 6, 7, 8, and 9 tend to have better RFS and they also have higher cytokine expression when stimulated with NY-ESO-1

At week 6 (after 2 cycles of ipilimumab), 15 cytokines were found to have higher expression when stimulated with NY-ESO-1 versus gp-100 or MART-1. On stimulation with NY-ES0-1, expression of IL-1β, VEGF, G-CSF, HGF, IL-13, IL-17, GM-CSF, MCP-1, IL-5, IL-7, IL-4, IL-10, IFN-γ, IL-8 and IL-2 was significantly higher (p value <0.05 for each) (Fig. 2).

### Cytokine expression levels at baseline and at week 6 correlate with recurrence free survival (RFS)

Patients were clustered into two groups based on cytokine expression profile in response to NY-ESO-1 vs gp100 and MART-1. At baseline, patients 1, 5, 6, 7, 8, 9 and 10 were in cluster 1, and patients 11, 17, 18, 20, 22 and 28 were in cluster 2. The cluster result was correlated with RFS at 12 months. Poor RFS was defined as patients having died or relapsed within 1 year, and good RFS meant patients had not died or relapsed within 1 year. Though there was no statistically significant association between cluster assignment and dichotomized RFS (p = 0.10), there was a trend towards improved RFS in cluster 1, where 5 out of 7 patients had good RFS. In cluster 2, 5 out of 6 patients had poor RFS.

At week 6, patients 1, 5, 6, 7, 8, and 9 were in cluster 1, and patients 10, 11, 17, 18, 20, 22, and 28 were in cluster 2. 4 out of 6 patients in cluster 1 had good RFS and 5 out of 7 patients in cluster 2 had bad RFS, however, there was no statistically significant association between cluster assignment and dichotomized RFS (p = 0.29).

**Fig. 3:**
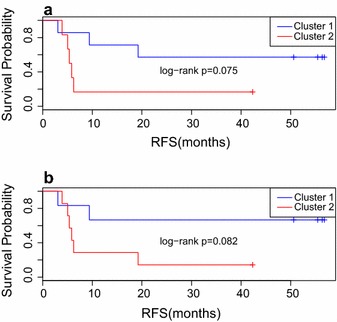
RFS by clustering; clustered by baseline cytokine levels:* Panel* **a** RFS in cluster 1 versus cluster 2, where patients are clustered by cytokine expression profile at baseline.* Panel* **b** RFS in cluster 1 versus cluster 2, where patients are clustered by cytokine expression profile at week 6

We also observed differences in RFS based on cytokine level clustering both at baseline (p = 0.075) and at week 6 (p = 0.082) (Fig. 3).

## Discussion

Induction of potent antitumor T cell immunity is considered to be crucial for the therapeutic efficacy of immunotherapy. Here, we report on cytokine expression profiles from the PBMCs of 13 patients treated with neoadjuvant ipilimumab at baseline and at week six. We examined responses to two melanoma lineage (gp100, MART-1) and a cancer testis (NY-ESO-1) antigen, all commonly expressed by the majority of melanoma tumors [11–13] and all known to be spontaneously immunogenic. We chose to assess expression levels of cytokines and chemokines with widely varied functions, and we show that at baseline (prior to treatment with ipilimumab), cytokine profiles are differentially expressed in response to antigen stimulation. We find that PBMC supernatants express 17 cytokines at higher levels when stimulated with NY-ESO-1 versus gp100 or MART-1, in line with the known spontaneous immunogenicity of NY-ESO-1 in eliciting CD4 and CD8 T cell responses [14] in addition to antibody responses. On stimulation with NY-ESO-1, expression of IL-1β, MIP1-β, IL1-RA, VEGF, IL-13, IL-17, MIP1-α, GM-CSF, MCP-1, IL-5, IL2-R, IL-4, IL-10, IFN-γ, TNF-α, IL-8 and IL-2 was significantly increased. Interestingly, these cytokines fall into varying functional groups, including pro-inflammatory (IL-1β, MIP-1β, IL-17, IFN- γ, TNF-α and IL-8) and anti-inflammatory (IL-1RA, IL-10). We also find TH1 (IFN-γ, IL-2,) and TH2 (IL-4, IL-13) cytokines. These findings suggest that PBMCs of patients with advanced melanoma are polyfunctional, with expression of cytokines across varied pathways.

NY-ESO-1 is a member of the cancer testis antigen (CTA) family, a group of tumor-associated antigens that are expressed on multiple different tumor types, with expression in normal tissues restricted to placenta and testis [15]. NY-ESO-1 mRNA is expressed in 17–42% of melanomas [15–17], and it is thought to be the most immunogenic CTA [15]. Up to 50% of patients with advanced melanoma whose tumors express NY-ESO-1 are seropositive for anti-NY-ESO-1 antibodies [18], and even in the absence of detectable antigen expression, NY-ESO-1 may induce NY-ESO-1 antibodies in 10% of patients [19]. NY-ESO-1 has also been shown to stimulate spontaneous and synchronized humoral and cell-mediated immune responses. In one study, more than 90% of patients with circulating anti-NY-ESO-1 antibodies also had an NY-ESO-1-specific CD8^+^ T-cell response, whereas this effector T cell response was absent in patients without detectable anti-NY-ESO-1 antibody [20]. Therefore, many clinical trials of vaccines employing NY-ESO-1 are ongoing, with several already reported [21–24]. Thus, detection of multiple cytokines at elevated expression levels in response to NY-ESO-1 antigen stimulation suggests that patients with advanced disease (in our cohort, 92% had stage IIIC melanoma) have an NY-ESO-1 specific immune response, though this is likely functionally dampened in the tumor microenvironment by immune checkpoints such as PD-1/PD-L1 [25] and CTLA-4 [26].

When PBMC cytokine expression levels were tested at week 6, after 2 cycles of ipilimumab, we find that 15 cytokines have increased expression when stimulated with NY-ESO-1 versus gp-100 or MART-1. Specifically, expression of IL-1β, VEGF, G-CSF, HGF, IL-13, IL-17, GM-CSF, MCP-1, IL-5, IL-7, IL-4, IL-10, IFN-γ, IL-8 and IL-2 was significantly increased. Increased expression in response to NY-ESO-1 versus gp-100 or MART-1 at week 6, as we saw at baseline, again suggests increased activated cell specificity for the NY-ESO-1 antigen. In addition, we again note cytokine and chemokine expression across multiple effector functions. Five proteins were elevated at baseline in response to NY-ESO-1 antigen, but not at week 6: IL-2R, IL-1RA, MIP-1α, MIP-1β, and TNF-α. At week 6, we noted an increase in G-CSF, IL-7 and HGF, which had not been noted at baseline. Though difficult to draw conclusions from this small cohort, an increase in growth factors may indicate increased CD4 and CD8 T cell activation, though G-CSF is a key modulator of MDSC [27, 28], and G-CSF expression has correlated with increased MDSC and worse overall survival in murine models [29].

We had previously reported that in 24 patients treated with neoadjuvant ipilimumab, for whom tumor tissue was available, CD8 + tumor infiltrating lymphocytes were increased after treatment [10]. Furthermore, a significant increase in circulating CD4^+^, CD8^+^ and T regulatory cells (Treg; CD4^+^ CD25^+^ Foxp3^+^) was noted at week 6, compared to baseline, and this correlated with improved PFS [10]. Numerous subsets of Treg have diverse functions in relation to the anti-tumor responses [30], and our findings of higher pro- and anti-inflammatory cytokines in response to antigen stimulation with NY-ESO-1 after 6 weeks of treatment suggests that ipilimumab does not only function by a pro-inflammatory mechanism. Moreover, the pro-inflammatory response appears to be the most dominant, based on increases in several pro-inflammatory cytokines, including IL-1β, IL-6, MIP-1α, IL-17, IL-8, IL-2, TNF-α and IFN-γ, and this may be the mechanism leading to the clinical benefit seen in a subset of patients. In an exploratory analysis, we assessed the association between Treg and MDSC with our cytokine data, and no significant associations were found after adjusting for multiple testing, likely due to the small sample size that we are reporting on. We clustered patients at baseline and at week 6 into two groups based on their cytokine expression profile. At baseline, seven patients were in cluster 1 and had higher cytokine expression when stimulated with NY-ESO-1, while six patients were in cluster 2. Poor RFS was defined as patients who had died or relapsed within 1 year, whereas patients with good RFS had not died or relapsed within 1 year. Notably, the cluster into which patients were placed based on their cytokine expression profile was found to correlate with RFS at 12 months. While this did not reach statistical significance, there was a sharp trend towards improved RFS in cluster 1, where 5 of 7 patients had good RFS, whereas in cluster 2, 5 of 6 patients had poor RFS. These differences in RFS based on cytokine level clustering were noted both at baseline and at week 6. This suggests that having higher cytokine expression in respond to NY-ESO-1 compared to gp-100 and MART-1 may have some association with better RFS.

Our study has several limitations. The small cohort size limits the conclusions that can be drawn, and may be why we do not see a statistically significant difference when we compare RFS in cluster 1 versus cluster 2. Furthermore, the cytokine expression levels were determined in supernatants of PBMC, clearly limiting our ability to differentiate between expression profiles of specific cell subsets. Our earlier study testing intracellular cytokine expression confirmed that both CD4^+^ and CD8^+^ T cells produced IFN-γ, but many of these circulating proteins have other sources. To our knowledge, this is the first study to report on cytokine expression profiles from PBMCs of patients treated with ipilimumab. Furthermore, we compare levels at baseline and after 6 weeks of treatment. We show that at baseline, 17 cytokines are expressed at higher levels in response to NY-ESO-1 than in response to gp-100 or MART-1. We show that this difference is maintained after 6 weeks of treatment of ipilimumab. Importantly, we find that when patients are clustered into groups based on their cytokine expression profile, patients in cluster 1 had superior RFS compared to patients in cluster 2. These findings warrant validation across a larger sample size, and it would also be important to determine if these differences are also seen in patients with metastatic melanoma who are treated with ipilimumab. If our findings persist in larger cohorts, this may help determine which patients are likely to derive RFS benefit from ipilimumab.

## Conclusion

Here, we report on cytokine expression profiles of 13 patients treated with ipilimumab in the neoadjuvant setting. The supernatants collected from the PBMCs of patients treated with ipilimumab had increased cytokine expression when stimulated with NY-ESO-1 in comparison to gp-100 or MART-1. Interestingly, these cytokines belong to a broad functional range, including inflammatory, type 1, type 2 and regulatory functions, and warrant further study. Furthermore, patients whose PBMCs secreted more cytokines, most notably in response to NY-ESO-1, tended to have better RFS. This suggests that cytokine expression profiles may have predictive value in the context of CTLA-4 blockade that warrant further exploration.

## Additional file

**Additional file 1.** Summary of Luminex cytokine measurements.

## Abbreviations

CTA: cancer testis antigen
CTLA-4: cytotoxic lymphocyte antigen 4
ECOG: Eastern Cooperative Oncology group
IL: interleukin
IFN: interferon
Ipi: ipilimumab
MDSC: myeloid derived suppressor cells
PBMC: peripheral blood mononuclear cells
RFS: relapse free survival
Treg: regulatory T cells
