# Supplementary material for: Melanoma antigen-specific effector T cell cytokine secretion patterns in patients treated with ipilimumab
Source: J Transl Med. 2017 Feb 21;15:39. doi: 10.1186/s12967-017-1140-9 (PMC5319167; doi:10.1186/s12967-017-1140-9)
Supplement: Supplementary file 1 — Additional file 1. Summary of Luminex cytokine measurements. [file 12967_2017_1140_MOESM1_ESM.docx]

Summary of Luminex Cytokine Measurement

The tables summarize the median and range of cytokine levels measured under the five conditions in the unit of pg/mL. A value lower than the detection limit was assigned the lower limit value (LLD value) if it is measured under positive or negative control, and was assigned zero otherwise.

## Baseline Measurement

|  | **PBMC ALONE** | **+PMA/ION** | **+MART-1 PEPTIDE POOL** | **+gp100 PEPTIDE POOL** | **+NY-ESO-1 PEPTIDE POOL** |
| --- | --- | --- | --- | --- | --- |
| IL.1b | 5 (5-5) | 5 (5-5) | 0 (0-0) | 0 (0-0) | 0 (0-47) |
| FGF.basic | 10 (10-18) | 13 (10-18) | 0 (0-15) | 0 (0-14) | 11 (0-22) |
| IFN.a | 10 (10-10) | 10 (10-25) | 0 (0-0) | 0 (0-15) | 0 (0-28) |
| Exotaxin | 1 (1-1) | 1 (1-1) | 0 (0-0) | 0 (0-0) | 0 (0-0) |
| MIP.1b | 40 (9-62) | 65 (15-2209) | 38 (11-57) | 52 (11-98) | 208 (27-5501) |
| IL.1RA | 109 (60-155) | 299 (158-1052) | 128 (54-207) | 109 (18-633) | 236 (108-1046) |
| VEGF | 1 (1-1) | 1 (1-3) | 0 (0-0) | 0 (0-0) | 0 (0-4) |
| G.CSF | 5 (5-12) | 8 (5-22) | 0 (0-9) | 0 (0-17) | 8 (0-12) |
| EGF | 2 (1-7) | 2 (1-15) | 0 (0-8) | 2 (0-9) | 2 (0-7) |
| HGF | 5 (5-8) | 8 (5-18) | 0 (0-8) | 0 (0-12) | 5 (0-22) |
| IL.12 | 22 (9-53) | 32 (16-50) | 19 (10-41) | 21 (7-53) | 25 (11-70) |
| RANTES | 369 (159-1463) | 654 (310-1834) | 494 (210-1103) | 431 (96-1741) | 419 (221-2847) |
| IL.13 | 1 (1-1) | 1 (1-46) | 0 (0-0) | 0 (0-0) | 0 (0-153) |
| IL.15 | 5 (5-17) | 5 (5-39) | 0 (0-19) | 0 (0-23) | 0 (0-9) |
| IL.17 | 1 (1-1) | 1 (1-19) | 0 (0-0) | 0 (0-0) | 0 (0-67) |
| MIP.1a | 17 (5-28) | 71 (7-3517) | 19 (0-29) | 41 (0-165) | 162 (13-5496) |
| GM.CSF | 1 (1-1) | 1 (1-46) | 0 (0-0) | 0 (0-0) | 0 (0-59) |
| MCP.1 | 108 (55-310) | 297 (39-2632) | 155 (68-410) | 211 (39-708) | 400 (98-4774) |
| IL.5 | 1 (1-1) | 1 (1-10) | 0 (0-0) | 0 (0-0) | 0 (0-97) |
| IL.7 | 10 (10-10) | 10 (10-19) | 0 (0-0) | 0 (0-22) | 0 (0-37) |
| IP.10 | 3 (3-5) | 6 (4-11) | 4 (2-7) | 4 (1-8) | 5 (3-7) |
| IL.2R | 26 (15-70) | 55 (15-386) | 39 (0-76) | 26 (0-45) | 111 (0-503) |
| MIG | 1 (1-1) | 1 (1-1) | 0 (0-0) | 0 (0-0) | 0 (0-0) |
| IL.4 | 6 (3-12) | 6 (3-87) | 4 (3-12) | 6 (3-12) | 11 (4-318) |
| IL.10 | 1 (1-1) | 1 (1-18) | 0 (0-0) | 0 (0-0) | 0 (0-24) |
| IL.6 | 4 (2-9) | 7 (4-53) | 5 (2-9) | 7 (0-15) | 5 (2-41) |
| IFN.g | 1 (1-1) | 1 (1-133) | 0 (0-0) | 0 (0-0) | 0 (0-175) |
| TNF.a | 2 (1-3) | 4 (1-294) | 2 (0-4) | 2 (0-7) | 9 (0-542) |
| IL.2 | 1 (1-1) | 1 (1-1160) | 0 (0-0) | 0 (0-0) | 0 (0-2021) |
| IL.8 | 883 (400-2145) | 5149 (1236-20926) | 1092 (568-2203) | 1286 (157-22000) | 2100 (785-22000) |

## Week 6 Measurement

|  | **PBMC ALONE** | **+PMA/ION** | **+MART-1 PEPTIDE POOL** | **+gp100 PEPTIDE POOL** | **+NY-ESO-1 PEPTIDE POOL** |
| --- | --- | --- | --- | --- | --- |
| IL.1b | 5 (5-5) | 5 (5-12) | 0 (0-0) | 0 (0-0) | 0 (0-10) |
| FGF.basic | 10 (10-10) | 10 (10-18) | 0 (0-20) | 0 (0-14) | 0 (0-18) |
| IFN.a | 10 (10-10) | 10 (10-15) | 0 (0-0) | 0 (0-0) | 0 (0-28) |
| Exotaxin | 1 (1-1) | 1 (1-1) | 0 (0-0) | 0 (0-0) | 0 (0-0) |
| MIP.1b | 26 (11-95) | 119 (12-2672) | 30 (6-101) | 41 (14-102) | 49 (6-3657) |
| IL.1RA | 77 (20-197) | 236 (108-1161) | 77 (27-270) | 197 (45-398) | 187 (0-1046) |
| VEGF | 1 (1-1) | 1 (1-4) | 0 (0-0) | 0 (0-1) | 0 (0-2) |
| G.CSF | 5 (5-8) | 5 (5-17) | 0 (0-12) | 0 (0-8) | 8 (0-17) |
| EGF | 1 (1-4) | 1 (1-6) | 0 (0-4) | 0 (0-8) | 0 (0-6) |
| HGF | 5 (5-10) | 5 (5-15) | 5 (0-8) | 0 (0-12) | 10 (0-14) |
| IL.12 | 19 (5-63) | 28 (11-60) | 20 (0-55) | 27 (6-46) | 25 (0-66) |
| RANTES | 327 (51-2498) | 516 (153-3025) | 384 (74-1856) | 581 (106-1108) | 498 (71-1546) |
| IL.13 | 1 (1-1) | 1 (1-91) | 0 (0-0) | 0 (0-0) | 0 (0-33) |
| IL.15 | 5 (5-5) | 5 (5-30) | 0 (0-8) | 0 (0-10) | 0 (0-17) |
| IL.17 | 1 (1-1) | 1 (1-100) | 0 (0-0) | 0 (0-0) | 0 (0-14) |
| MIP.1a | 11 (5-46) | 119 (11-3732) | 17 (0-65) | 28 (0-154) | 36 (0-4251) |
| GM.CSF | 1 (1-1) | 1 (1-88) | 0 (0-0) | 0 (0-0) | 0 (0-41) |
| MCP.1 | 119 (34-657) | 263 (26-2678) | 138 (39-814) | 135 (36-731) | 464 (51-2650) |
| IL.5 | 1 (1-1) | 1 (1-52) | 0 (0-0) | 0 (0-0) | 0 (0-10) |
| IL.7 | 10 (10-10) | 10 (10-15) | 0 (0-0) | 0 (0-0) | 0 (0-32) |
| IP.10 | 4 (1-5) | 5 (3-8) | 3 (2-6) | 4 (2-7) | 5 (0-8) |
| IL.2R | 15 (15-49) | 64 (15-448) | 32 (0-64) | 39 (0-82) | 58 (0-435) |
| MIG | 1 (1-1) | 1 (1-1) | 0 (0-0) | 0 (0-0) | 0 (0-0) |
| IL.4 | 6 (3-12) | 6 (3-197) | 6 (3-12) | 5 (0-12) | 11 (0-64) |
| IL.10 | 1 (1-1) | 1 (1-51) | 0 (0-0) | 0 (0-0) | 0 (0-9) |
| IL.6 | 2 (1-15) | 5 (1-41) | 3 (0-22) | 6 (0-22) | 5 (0-50) |
| IFN.g | 1 (1-1) | 1 (1-191) | 0 (0-0) | 0 (0-0) | 0 (0-120) |
| TNF.a | 1 (1-9) | 9 (1-580) | 2 (0-15) | 3 (0-10) | 2 (0-620) |
| IL.2 | 1 (1-1) | 1 (1-1601) | 0 (0-0) | 0 (0-0) | 0 (0-709) |
| IL.8 | 504 (188-3529) | 2752 (575-22000) | 618 (447-2762) | 1759 (432-10539) | 1757 (336-22000) |
